# The Construction and Government of Lunatic Asylums and Hospitals for the Insane

**Published:** 1847-07-01

**Authors:** 


I. The Construction and Government of Lunatic Asylums and Hospitals for the Insane. By *John Conolly*, M.D. 12mo. pp. 183. Churchill, 1847.

II. Journal of Insanity, Vol. III. Utica, U. S. 1846.

III. Third Annual Report of the Managers of the State Lunatic Asylum. By *Amariah Brigham*, M.D. Albany, U.S. 1846.

IV. Twenty-Second Report of the Officers of the Retreat for the Insane at Hartford, Conn., U.S. 1846.

V. Report of the Medical Officers of the Lunatic Asylum for the County of Lancaster. 1846.

Although we have recently* treated somewhat at large upon the subject of Insanity, yet so important is the topic, that we are glad to avail ourselves of every additional opportunity of adverting to it, whether to chronicle progress or to indicate the obstacles to this. All that we have seen or read since they were written but confirms the correctness of the opinion we advanced in the articles referred to, that the government of Lunatic Asylums in this country is founded upon an essentially faulty basis, and that, until this error is remedied, the management of the Insane will not continue to be such as is most conducive to their interests. At present this is a matter of the merest chance, dependent upon the amount of intelligence, good sense and forbearance possessed by so miscellaneous a class of persons, as far as mental attributes and special information are concerned, as the visiting justices and governors of these establishments. From Dr. Conolly's work we incidentally learn the mischievous powers possessed and employed by them at Hanwell, and certain it is that it required an amazing union of humanity, patience, discretion, and firmness upon the part of this philanthropist to carry his great experiment to a successful issue. Few men are equally endowed, and thus we see, as a natural effect of the interference and irritating annoyances, they are subject to a constant change of the resident medical officers in our various asylums. So peculiar is the condition of the lunatic, that we do not hesitate to repeat our opinion, that his real well-being, whether rich or poor, can only be provided for effectually by his becoming an object of direct state-interference or management: but, supposing that the prejudices against any such centralizing procedure would prevent its adoption, we may at least imitate the Americans, who have the good sense to leave the entire management of their asylums, at least of all that relates to the patients, exclusively in the hands of a well-selected and well-paid resident medical superintendent. Until an officer of this kind is allowed absolute control over it, no establishment can be considered in a satisfactory condition.

* Vide, Med. Chir. Rev. N.S., Vol. IV. pp. 56 and 394.

He is the central point around which all must hinge and turn. Take every imaginable pains in his selection, but having chosen him remunerate him handsomely, and clothe him with the necessarily despotic authority. The present humiliating position of most of our resident officers, as contrasted with that of their brethren on the other side of the Atlantic, is as distressing as it is mischievous.

Dr. Conolly's little work consists chiefly in a reprint of some Lectures which appeared in the Lancet last year, as supplementary to his valuable clinical course which we had hoped would also have been re-published. It is full of matter of the highest importance, especially at this epoch, when so many new establishments for the reception of the insane are upon the eve of erection. We may notice some of the chief topics he adverts to; but we may here observe that, we do not participate in the fears which he entertains that the projected separation of the chronic from the more recent cases will be attended with the diminution of the comforts or attentions at present enjoyed by the former, at all events we are certain that it need not be so. While we acknowledge that each county is bound to provide suitable establishments for all its lunatics, it seems only reasonable that such provision should be made with all the view to curability and economy that the comfort of the patients admits of.

*Site and Construction of Asylums.*—Dr. Conolly properly insists that, any plan for the construction or alteration of a lunatic asylum, should be submitted to the consideration of a physician well acquainted with the characters, habits, and wants of the insane prior to its final adoption, he frequently being best able to point out the defects, or to suggest improvements. Heretofore, asylums had too much the appearance of prisons, and in point of fact, both descriptions of edifice were constructed for merely the safe detention of their inmates. A great improvement in this respect has taken place in the case of recently-erected asylums, and we hope ere long none of the establishments so destined will retain the repulsive and forbidding appearance which some yet have. Dr. Conolly lays much stress upon a gentle eminence being chosen for the site; and all those who have seen the beautiful situation of the Lincoln Asylum, and are aware of the generally healthy state of its inmates and the happiness which the extended prospect imparts to them, will be disposed to agree with him. He thinks the building should not be constructed for more than 360 or 400 patients, great care being taken to enclose sufficient space within the walls to furnish ample gardens, airing courts, and to admit of the due classification and employment of the insane, and of the enlargement of the building, if necessary, without violating the original plan. In all asylums the proportion of separate sleeping-rooms is too small, and Dr. Conolly always recommends that at least two-thirds of the patients should be so accommodated. With large overcrowded dormitories a sweet state of the air cannot be maintained. Whatever system of ventilation and warming be adopted, Dr. Conolly lays great stress upon the necessity of having windows large enough to admit an abundant supply of fresh air in temperate weather, and open fire-places, with all their cheering associations in Winter. He insists, too, upon having the galleries well-lighted, for not only is it an error to suppose that the insane are benefited by being kept in a state of darkness or semi-darkness ; but they suffer grievously when condemned to this from motives of economy. " In visiting patients in private houses," he observes, " I generally find the rooms made totally dark, but filled with anxious relatives, attendants, and half the servants of the house. All these things are unfriendly to the patient's tranquillity, and produce suspicion, fear, and increased violence." The maintenance of the most perfect cleanliness, both of the persons and the chambers of the insane, seems so obviously of the first importance, that one is surprised to find it is one of the essentials only quite recently and not yet universally recognized. It is one, indeed, impossible to put into force by other means than a well-organized superintendence.

*Exercise and Recreations.*—We sincerely hope that, in any new establishment that may be erected, ample provision will be made for out-of-doors exercise, in which most of our public asylums, and nearly all the private ones, are terribly deficient. The sight of hundreds of restless lunatics cooped up within a miserable space of ground, encompassed by high walls, is one of the most mournful of spectacles, and the wonder is that reason is ever restored under such discouraging circumstances. Even in our best asylums, the curative, ameliorating, and composing effects of exercise are not developed, by reason of the rules which prevent the patients passing beyond the walls. Large, indeed, must be the space enclosed if traversing it again and again for months and years do not prove insupportably monotonous. In America, all the patients who can be depended upon are permitted, with the best effects, to take walks and excursions into the adjoining country, and why the same practice should not be followed here it would be difficult to say. The various out-of-door amusements should be encouraged, as far more useful than those of a more sedentary character, and as acceptable to the attendants, who stand as much in need of relaxation, as well as to the patients. Within-doors, the means of amusement are now freely resorted to in most asylums ; and, indeed, since attention has been drawn to the necessity of providing a variety of such as a substitute for restraint, some have been introduced into some of the foreign asylums, such as dramatic representations, debating societies, &c., of questionable utility. Upon the defective condition of the majority of private asylums in regard to exercise, Dr. Conolly observes as follows :—

" It is astonishing to find that one of the particulars of treatment in which the rich patient is sometimes more unfavourably situated than the poor, is that of having an opportunity of enjoying free and frequent exercise. The untrodden lawn, the dusty and desolate courts, the paved yards, the wretched sheds, the lonely out-houses, together with the closed doors and windows of a private asylum, often give to such places an external character, which makes the visitor regard them with dread, and the passer-by speak of them in whispers. The idea of their entrance is connected with that of the fatal gate, which whoso entered left all hope behind. If a patient is visited in such a house, the unlocking of doors, the threading of passages, and the ascent of gloomy stairs, with the close atmosphere of apartments, filled with patients sitting by the walls, oppressed with indolence and monotony, are all features too familiar to those who knew such houses before better improvements penetrated into them. And it is only in a few of the best private asylums that we even now find cheerful sitting-rooms, opening into gardens, into which patients may walk when they please ; and the benefit of this improvement is yet too generally limited to the most rational of the patients, and not extended to the irritable and troublesome, who ought also to be able to go out of their sitting-rooms, although into gardens more secluded and secure." P. 59.

Dr. Conolly believes that attention to the *dress* of patients is too much neglected, and all those who have seen the humiliated mien of some lunatics when presented to friends or strangers in unsuitable attire, will allow that this may act very detrimentally upon them.

"Many private asylums are open to the charge of great neglect as respects the dress of the classes far above pauperism. Tattered and thread-bare coats, very shabby hats, trowsers not always free from an offensive smell; and equally slovenly dresses on the female side of the asylum, shoes out of repair, hair in curl-papers, make the unfortunate patients objects of pity or of ridicule. They feel themselves degraded, lose their self-respect, and with it the little self-control their malady has left them. It is very true that, in some cases, the patients will not dress themselves properly, that they have an affection for old and ragged garments, insist upon their being fit to go to court in, and are evidently offended if better clothes be substituted for them; but such cases only form a small proportion in any asylum; and, in many instances, habits of personal neatness may be long preserved, and in some restored, after being long lost." P. 63.

*Employment,*—The value of this can scarcely be over-estimated, when discriminately furnished. "We consider labour," says Dr. Brigham in his Report, "as among the most essential of our curative means: of this we become more convinced every year." It should, however, never be regarded apart from the other portions of the treatment of the patient, and its amount therefore regulated by the opinion of the physician, not by its economical results, important though these be in large establishments. Out-of-doors employment is far more conducive to health and recovery than that of a sedentary character. Regular work should never be attempted to be enforced from an unwilling patient upon the plea of idleness. According to the last Hanwell Report, 219 out of 418 male patients were employed, and 314 out of 567 women. All the patients' clothing, except shoes, and bedding is made up in the asylum, and all the washing and mending for so large a population performed. There are a garden and farm, on which labour is performed, carpenter's, tailor's, shoemaker's, tinman's, &c. shops, the expense of employing instructors being an obstacle to the multiplication of occupations. Dr. Brigham complains also of this, and that in a much smaller establishment than Hanwell.

"In Winter, we find it difficult to furnish suitable and sufficient labour for all who would be benefited by it. Some engage in sawing wood, but this furnishes but little labour for 60 or 70 men. Some work in the joiner's shop, and a few are employed in other kinds of business. Still, for a considerable number we have not sufficient employment, and we apprehend this is a difficulty which all large asylums have to encounter. We have studiously examined this subject, and reflected much upon the propriety of establishing some kind of business, in which many can engage. But we find it difficult to determine what kind is best. Hitherto, we have found none better than *carving toys* and making small wooden articles. Several of our patients have become very skilful at this business. Some articles carved here are not inferior to the handsomest Swiss specimens, of which in fact ours are imitations." P. 33.

The difficulty becomes greater still in devising occupation for male patients in the upper walks of life.

*Attendants.*—Upon no one circumstance does the well-doing of an asylum so essentially depend as upon the possession of an efficient corps of attendants. The securing this is of the first consequence then, for without it no ameliorations can be carried out to the extent they are capable of, and no improvement can be prevented from eventual degeneration. Two chapters are therefore devoted by Dr. Conolly to this important subject, in which he points out the defects of the present modes of appointing and governing these functionaries, and suggests some of the numerous alterations these are capable of, and urgently demand. It is so evident that these persons should be the faithful and passive instruments in the hands of the physician for carrying out his designs, that it is scarcely to be believed that they are chosen without reference to his wishes or their own qualifications, called upon to perform duties of a nature and at a time that have not his sanction, and dismissed or changed with as little consideration of his feelings upon the subject or the true welfare of the patients as prevailed in their appointment! We are constantly hearing complaints of the unfit class of persons who follow the occupation of keepers or attendants of the insane, and can it be wondered at that such should be the case when our large asylums set so vicious an example as this. The Resident alone can appreciate their qualifications and their efficiency; to him alone should they owe their introduction to the establishment and their continuance in it; or at the very least he should possess a *veto* upon the one and the other. Nothing is more admirable than the spirit of benevolence which pervades the whole of Dr. Conolly's work, and this is not exhausted upon the insane themselves; for, while he demands that steps be taken to provide them with more efficient attendants, he likewise insists that these ought to be more considerately treated in regard to their distribution of employment, relaxation and recreation, instruction, &c. With the present hard work and uncertain tenure of an asylum-assistant, no person will longer engage in the occupation than he can help, those best qualified for it indeed being the first to quit it, leaving the staff overburdened with the unwilling and the incapable. The duties to be performed at Hanwell, here described in full detail, are indeed multifarious and onerous, and make so large a call upon the benevolence, the mental energy, and the bodily activity of the attendants, that we are at a loss to understand how they can be efficiently executed by persons so miscellaneously chosen and so inadequately encouraged. Dr. Brigham says that, in his asylum, great advantage has been derived from a more judicious distribution of duties among the attendants, so as occasionally to exempt them from duties which, uninterruptedly continued for many hours, must produce a tension of mind utterly destructive to the retention of its elasticity and detrimental to the preservation of the temper. These supplementary attendants are derived from a class of persons sufficiently instructed to develop the amount of mental occupation the insane are capable of and benefited by.

" Our observation for many years in various lunatic asylums, led us a long time since to regard the want of *mental occupation* as the greatest want in modern institutions for the insane. Go into any such establishment, and you will find some few, in Winter a very few, at work, some playing cards or other games; yet a still larger class will be found sitting about, listless, inactive, doing nothing, saying nothing, taking no interest in anything going on around them, gathering round the stove, looking forward to nothing but the hours for eating and retiring to sleep. For a short time each day, when the physician passes round, they will exhibit a little animation and say a few words, and then relapse into their former condition.

"These patients give but little trouble in an asylum, and are very apt to be overlooked and neglected, and, if not already demented, soon become so. They are thought not to require much attention, as they have good bodily health, and are quiet, consequently they generally receive but little notice. *But this class require great attention.* They need *mental exercise;* they should attend school, and have their minds aroused into mental activity for an hour or two every day. Soon, by this course, their memories will improve, they will become interested in singing or study, and by perseverance some will be cured, and many, very many, rendered capable of much enjoyment, and be kept from sinking into a state of hopeless dementia. * * * *

"Our teachers spend all their time with the patients, but have no labour nor any other duty to attend to, than to interest the patients, and contribute all they can by their presence and conversation to their contentment and enjoyment. Thus they join in their amusements and walks, and are their constant companions. The relief which they afford the attendants is very great, and enables us to dispense with some that would be otherwise necessary. We are satisfied that an establishment like this can be better managed, and with equal economy, by having an arrangement by which some should devote their time to their ordinary duties and labours of the halls, while others should have nothing to do but to accompany the patients and endeavour to instruct and amuse them. The latter having nothing to do with any coercive measures, the patients do not become prejudiced against them, and will readily hearken to their suggestions. Thus they serve as a constant guard, and by their presence and management prevent outbreaks and disorder, and make coercive measures, restraint, and seclusion rarely necessary.—*Report*, p. 37.

There seems sound wisdom in this arrangement, both as regards the welfare of the insane and the comfort of the attendants; and it would be still more applicable to the pauper lunatics of England than to those of America, inasmuch as a much smaller proportion of the former are able to read, and thus amuse themselves by the perusal of light-reading, newspapers, &c., with which, too, our asylums are far more scantily provided. But the lack of well-informed attendants is most felt by the wealthy and educated lunatic, who finds himself condemned to the perpetual society of one possessed only of the acquirements of a menial, but invested with the authority of a master, and that too often a harsh one. Little matters it that a ray of intelligence may yet lurk amidst the ruins of his mental edifice, no sympathy and encouragement will enkindle it into an invigorating flame; and the mental apathy and listlessness which an utter absence of interesting conversation or other intellectual occupation engenders or augments, may confirm and render hopeless a condition which, more skilfully managed, might have had another issue. When it is considered how many persons there are of both sexes, whose mental acquirements and moral attributes would well fit them for the occupation of attending upon the insane, and the excellent remuneration the wealthy portion of so many of these unfortunates would enable them to furnish, it is evident that causes must exist for preventing persons who might be thus so mutually serviceable coming oftener into contact. The leading one is doubtless that indicated by Dr. Conolly, namely, the unintermitting attendance usually required at the hands of the attendant, no provision whatever being made for relaxation or temporary cessation of duties. Few minds possessed of the sensibility which constitute their most valuable property, could long withstand the harassing wear and tear incident upon their incessant occupation, or alternated with intervals of mere listlessness. Occasional breaking up of the routine of duties by the enjoyment of the society of the sane, healthful recreation, or change of occupation, is essential if the mind is to be maintained in its pristine vigour. It is evident that this cannot be accomplished as long as one individual alone has the charge of the insane patient; and we think it highly detrimental to the interests and comforts of the one and the other that he should ever be permitted to have this. Moreover, a person qualified as we have supposed would properly decline performing many menial offices now expected at the hands of the attendant. He should, therefore, always be provided with a subordinate for such purposes. This would entail an expense, not to be defrayed by other than the very wealthy, and would render the maintenance of the patient otherwise than in an asylum generally impossible. And we are convinced it is always undesirable he should be otherwise placed than under the roof of such an establishment; but we believe that the directors of this would find their account in employing a higher class of attendants, remunerating them better, and adjusting their duties more judiciously.

*Religious Services.*—Dr. Conolly, Dr. Brigham, and others of the most enlightened medical officers have frequently borne testimony to the value of these when subordinated to the other portions of the general plan, pursued with temperance and caution, and only to the extent sanctioned by the physician. Indeed, the good results which have attended their performance have been far greater *à priori* than might have been expected, seeing that so large a portion of the patients in the asylums of this country, and especially in America, have been originally rendered insane by perverted religious tenets and fanaticism. Still, every thing depends upon the prudence with which this instrument of improvement is wielded; for if it be placed in the hands of a rash intermeddling or conceited man, great evil will result. We quote some passages from a just appreciation of a Chaplain's various duties from the pen of Rev. T. Gallaudet, Chaplain of the Retreat at Hartford, U. S.

"So long as the insane have any exercise of their reasoning faculties left, and any moral and religious susceptibilities to be appealed to, (and no inconsiderable portion of them retain more or less of these faculties and susceptibilities, and some of them in a striking degree), so long Divine Truth, with its higher motives and consolations, will be found eminently adapted to the exigencies of their unfortunate condition, and one of the most salutary and efficacious means of cure. To what extent the influence of this Truth can be beneficially employed, time and a careful experience will show. Its effects should be critically noticed, and compared at different Institutions.

"In exhibiting these views, however, it ought to be stated, that the Chaplain by no means regards it the part of a wise performance of his duty, in his personal intercourse with the patients, to confine himself to conversation on religious topics merely. He endeavours with the general counsels, and often with the specific suggestions of the physician, to adapt the mode of this intercourse to the peculiar exigencies of the case. He appears among the inmates as their sympathizing friend. He exchanges with them the customary civilities of social life. He listens to their conversation, and lets them see that he is interested in it. He often introduces other than grave and serious subjects, adapted to afford rational instruction or innocent enjoyment; nor can he discover that, in doing this, he is exposed to any disparagement of the proper dignity of his office, by the want of courtesy or respect of those whom he seeks to benefit. It is, indeed, by pursuing such a course, that he hopes to avail himself of the suitable opportunities when they offer, and they not infrequently do offer, of presenting, in the most favourable manner, the simple and consoling truths of the Gospel. * * * *

"The good order, too, of such a numerous household, including the officers of the Institution, and others who are engaged in the management of its internal affairs; the conscientiousness, faithfulness, and kindness with which their various duties should be discharged; and the diffusion throughout the whole establishment of that spirit of self-denying benevolence which the Gospel teaches and inspires, are best promoted by constantly bringing before their attention, and commending to their cordial acceptance, as the rules of their conduct, the principles, the motives, and the encouragements contained in the oracles of Divine Truth."

A clerical officer actuated by these enlarged views must be an invaluable coadjutor to the physician, and is well deserving of the encomium passed upon him by Dr. Butler.

*Instruction.*—We can sympathize with the regret with which Dr. Conolly views the ill-judged suppression of the schools at Hanwell. Wherever they have been established in this country, in France, or in America, the testimony is universally in their favour as powerful means for ameliorating the condition of the patients. From the last Lancaster Report we extract the following.

"Exclusive of day-schools for the idiotic, an evening class for reading, writing, and arithmetic has been established in each ward, under the superintendence of the matron and chief-attendant, with such assistance as the ordinary attendant can bestow, aided in many cases by the better educated portion of the patients. A great number of the inmates take a lively interest in the proceedings, and in many, a marked improvement is observable. The day-schools are conducted on somewhat the same principle as that adopted in infant schools, and it is most gratifying to observe the favourable impression produced even on the idiotic mind by well-directed and persevering efforts, where, to the casual observer, all prospect of educational benefit would appear to be utterly hopeless."

Dr. Brigham, whose establishment possesses the great advantage of having attendants specially devoted to the instruction and amusement of the patients, says of the Schools:

"Our confidence in their utility has been increased by experience and observation. Many cases, we believe, cannot be improved, but by arousing and calling into exercise the dormant faculties of the mind. Hence we have found our schools particularly beneficial to the demented and those approaching that condition. In such, the active state of the disease, which originated the mental disturbance has passed, and left the brain and faculties of the mind in a torpid state. In these cases, medicine is in general of no use, and as we have said, they cannot often be much improved, but by exercising the faculties of the mind.

But others are also benefited by devoting a portion of every day to mental improvement. To those who are nearly or quite well, and who remain with us for fear of relapsing at home, or for other reasons, our schools afford enjoyment, and often means of improvement, which they highly value. Those that are uneasy and nervous, that are constantly restless and disposed to find fault and to annoy the attendants, and quarrel with all about them, because they have nothing else to occupy their minds, are also much benefited by the exercises of a school. We are every day surprised at the good effect they have upon this class of patients." P. 35.

Dr. Conolly concludes his work with some most excellent observations upon the proper officering of asylums; and ably illustrates the evils of the present plan of subdividing authority among the physician, matron, and governors. His advice, based upon long experience of suffering under the inconveniences he exposes, calls for the serious attention of all those who think that future attempts at ameliorating the condition of the insane should not be liable to risk of failure from intermeddling of well-meaning but ignorant lay-men, though these be "justices of the quorum."

Appended to Dr. Brigham's Report are some observations upon the *Prevention* of *Insanity.* He truly enough says that, if the predisposing causes were more sedulously avoided, that attacks and relapses of this dreadful disease would be far less frequent; and he seems to think that the public only need advising upon the liability of its being hereditarily transmitted or induced by faulty education and habits of life detrimental to health. Were this all, certainly to no one could we point as so capable of giving such advice as the learned author of the Influence of Mental Cultivation in inducing Insanity: but, alas! those who have to do with the realities of this bustling world, too soon become acquainted with the invincible carelessness or indifference of the public in respect to all hygienic precautions, and the too frequent impossibility or nullity of such amidst the increasing turmoils, struggles, and reverses of the present state of society. That insanity is upon the increase in this country, in France, in Germany and the United States can excite no surprise in the mind of those who are attentive spectators of what is passing around them.

With Dr. Brigham's opinion upon one other point touched upon by him we entirely agree, namely, that *medical men in general practice do wrong to neglect the study of insanity,* leaving it thus entirely in the hands of a special class of practitioners, and even rendering themselves incompetent to decide upon when the assistance of these can be most beneficially sought for. The earlier symptoms, are for this reason often overlooked or mistaken, and invaluable time lost, when by some overt act the patient makes patent to all the world that which the eye of science ought long since have provided for. Moreover, if preventive counsels are to be of any avail, they will be so by being specifically adapted to individual cases. You may furnish the public with the best popular treatises upon the subject, but they are unable or unwilling to correctly apply the principles these may contain. Our readers are aware that one of the highest claims Dr. Conolly has to the gratitude of the profession, is his earnest endeavours to diffuse information by his valuable clinical lectures at Hanwell.

We had intended citing one or two papers from the "Journal of Insanity," but have exhausted the space at our disposal.

